# Supplementary material for: CTCF and cohesin regulate chromatin loop stability with distinct dynamics
Source: eLife. 2017 May 3;6:e25776. doi: 10.7554/eLife.25776 (PMC5446243; doi:10.7554/eLife.25776)
Supplement: Supplementary file 2. — DOI: http://dx.doi.org/10.7554/eLife.25776.023 [file elife-25776-supp2.htm]

Supplementary File 2

Table with primer sequences

This table contains a list of primers used in
this study as well as a brief description of the experiment in which they were
used.

|  |  |  |
| --- | --- | --- |
| Name/description | Sequence (5’-3’) | Experiment |
| mESC Halo-mCTCF sgRNA 1: | TGAGGCGGTTGAAGCCATTG | Genome-editing (mESC C87 and C59) |
| mESC Halo-mCTCF sgRNA 2: | GGCGGTTGAAGCCATTGTGG | Genome-editing (mESC C87 and C59) |
| mESC Halo-mCTCF sgRNA 3: | ACTTACCAGAGACGCCGGGA | Genome-editing (mESC C87 and C59) |
| mESC Halo-mCTCF sgRNA 4: | GGCCCCCTTCCCGGCGTCTC | Genome-editing (mESC C87 and C59) |
| mCTCF genome F1 | AGCACTGGTAGTTCTTTGTGGT | Genotyping |
| mCTCF genome R1 | GTTGGCTTCGGAGGCTTCATA | Genotyping |
| Internal Halo F1 | GTCGCGCTGGTCGAAGAATA | Genotyping |
| mCTCF genome F2 | TAGCTTTCTAGTCATGCCCCAC | Genotyping |
| mCTCF genome R2 | AACCTCAGACAGCAGTCCTTC | Genotyping |
| Internal Halo R1 | GGGGTCGAATGGAAAGCCA | Genotyping |
| U2OS Halo-hCTCF sgRNA 1: | TGCAGTCGAAGCCATTGTGG | Genome-editing (U2OS C32) |
| U2OS Halo-hCTCF sgRNA 2: | TGATGCAGTCGAAGCCATTG | Genome-editing (U2OS C32) |
| U2OS Halo-hCTCF sgRNA 3: | AGTTTCGGACTCCTCCACAA | Genome-editing (U2OS C32) |
| U2OS Halo-hCTCF sgRNA 4: | ATAAAGGCAGGGGAAATGGA | Genome-editing (U2OS C32) |
| hCTCF genome F1 | ATCTTGCAGTGTTTCCCTCGG | Genotyping |
| hCTCF genome R1 | TGCCCTCCTCTGTATAACGC | Genotyping |
| hCTCF genome F2 | ACTGGTTCTCCCTCCAATCAGT | Genotyping |
| hCTCF genome R2 | ACGGTACCCACATAACGACCT | Genotyping |
| mESC mRad21-Halo/mRad21-SNAPf sgRNA 1: | CCTCAGATAATATGGAACCG | Genome-editing (mESC C45 and C59) |
| mESC mRad21-Halo/mRad21-SNAPf sgRNA 2: | CCACGGTTCCATATTATCTG | Genome-editing (mESC C45 and C59) |
| mESC mRad21-Halo/mRad21-SNAPf sgRNA 3: | ATCTAGCTCCTCAGATAATA | Genome-editing (mESC C45 and C59) |
| mRad21 genome F1 | ctggagcacccgtgacagttc | Genotyping |
| mRad21 genome R1 | CTGAGGAGTCACGCCACTGT | Genotyping |
| Internal SNAPf F1 | GAGAGCTTTACCCGCCAGG | Genotyping |
| mRad21 genome F2 | GAAGTGTCAAGCCAGGGGTA | Genotyping |
| mRad21 genome R2 | TGCGGTGCATAGCATCAGAG | Genotyping |
| Internal SNAPf R1 | CTGTTCGCACCCAGACAGTT | Genotyping |
| Nanog\_pos\_for | GCAGAGCCACAGAAGGAATC | ChIP-qPCR |
| Nanog\_pos\_rev | TTGCCACCTGAAACCACATG | ChIP-qPCR |
| Nanog\_neg\_for | CTTTGGACAGGCATCGTAGC | ChIP-qPCR |
| Nanog\_neg\_rev | GCAGTATCTCACCATGCAGC | ChIP-qPCR |
| chr2qC3\_pos\_for | AAGAACGCTGCTTTCATCCC | ChIP-qPCR |
| chr2qC3\_pos\_rev | ACAGCACCTAGTCACTCCTG | ChIP-qPCR |
| chr2qC3\_neg\_for | CACACTCCCCAAACACCAAC | ChIP-qPCR |
| chr2qC3\_neg\_rev | ACAACCTTAAATCCCTGGCTT | ChIP-qPCR |
| Ctcf\_mRNA\_for | CGGAACACAATGGCAAGACA | RT-qPCR |
| Ctcf\_mRNA\_rev | TTTCTACAGCAGGCTCCTCC | RT-qPCR |
| Rad21\_mRNA\_for | CTTCATGGTCTTCAGCGAGC | RT-qPCR |
| Rad21\_mRNA\_rev | GTCACTGTACGGCTCTTCCT | RT-qPCR |
| Pou5f1\_mRNA\_for | CCAGGCAGGAGCACGAGTGG | RT-qPCR |
| Pou5f1\_mRNA\_rev | CCTGGGACTCCTCGGGAGTTG | RT-qPCR |
| Sox2\_mRNA\_for | GAGTGGAAACTTTTGTCCGAGA | RT-qPCR |
| Sox2\_mRNA\_rev | GAAGCGTGTACTTATCCTTCTTCAT | RT-qPCR |
| Nanog\_mRNA\_for | CCTCAGCCTCCAGCAGATGC | RT-qPCR |
| Nanog\_mRNA\_rev | CCGCTTGCACTTCATCCTTTG | RT-qPCR |
| Esrrb\_mRNA\_for | GCACCTGGGCTCTAGTTGC | RT-qPCR |
| Esrrb\_mRNA\_rev | TACAGTCCTCGTAGCTCTTGC | RT-qPCR |
| Klf4\_mRNA\_for | CAGTGGTAAGGTTTCTCGCC | RT-qPCR |
| Klf4\_mRNA\_rev | GCCACCCACACTTGTGACTA | RT-qPCR |
| Zfp42\_mRNA\_for | TGTCCTCAGGCTGGGTAGTC | RT-qPCR |
| Zfp42\_mRNA\_rev | TGATTTTCCGACGTATGCAAA | RT-qPCR |
| Gapdh\_mRNA\_for | TGTGCAGTGCCAGCCTCGTC | RT-qPCR |
| Gapdh\_mRNA\_rev | TGAAGGGGTCGTTGATGGCAACA | RT-qPCR |
| Tfcp2l1\_mRNA\_for | CAGCCCGAACACTACAACCAG | RT-qPCR |
| Tfcp2l1\_mRNA\_rev | CAGCCGGATTTCATACGACTG | RT-qPCR |
